# How do Highly Sensitive Persons Parent their Adolescent Children? The Role of Sensory Processing Sensitivity in Parenting Practices

**DOI:** 10.1192/j.eurpsy.2026.11470

**Published:** 2026-07-31

**Authors:** A. Goldberg

**Affiliations:** Education & Psychology, Tel-Hai Academic College, Kiryat-Shmona, Israel

## Abstract

**Introduction:**

Individuals perceive and respond to their environment through the processing of sensory information. Dunn (1997) conceptualized sensory processing using the amount of stimuli needed for the nervous system to notice or react to stimuli (i.e., neurological thresholds) and the individual response in relation to the thresholds (i.e., behavioral responses). Sensory processing sensitivity (SPS) refers to an individual’s tendency to process more strongly and deeply a variety of information, including the arts, other peoples’ moods, hunger, and pain. An individual with a high measure of SPS is considered to be a highly sensitive person (HSP).

**Objectives:**

This research examines whether sensory processing sensitivity (SPS) in parents is associated with their parenting practices toward their adolescent children and whether attachment insecurity mediates the associations between SPS and parenting practices.

**Methods:**

One hundred twenty-one parent–adolescent dyads completed self-report questionnaires assessing parents’ SPS, parents’ adult attachment, and parenting practices.

**Results:**

Results showed that SPS was positively associated with inconsistency, psychological intrusiveness, and attachment anxiety. Further, attachment anxiety mediated the association between parents’ SPS and harsh parenting and partially mediated the association between parents’ SPS and parental psychological intrusiveness.

**Conclusions:**

There is very little research on how highly sensitive individuals parent their children in general and none regarding the parenting of high-SPS individuals during challenging developmental periods such as their children’s adolescence. Findings suggest that parents high in SPS may experience this period as especially stressful and high SPS might contribute to the use of negative parenting. Interventions focused on regulating high-SPS parents’ stress and on facilitating parents in practicing separating their own and their children’s emotions could promote their use of more positive parenting practices.

**Disclosure of Interest:**

None Declared

